# Modeling drug resistance in a conjoint normal-tumor setting

**DOI:** 10.1186/1742-4682-12-3

**Published:** 2015-01-15

**Authors:** Mitra Shojania Feizabadi, Tarynn M Witten

**Affiliations:** Physics Department, Seton Hall University, 400 South, Orange, NJ 07079 USA; Center for the Study of Biological Complexity, Virginia Commonwealth University, 1000 W. Cary Street, PO Box 842030, Richmond, VA 23284-2030 USA

**Keywords:** Cancer modeling, Cellular aging, Conjoint cell growth, Chemotherapy, Drug resistance

## Abstract

**Background:**

In this paper, we modify our previously developed conjoint tumor-normal cell model in order to make a distinction between tumor cells that are responsive to chemotherapy and those that may show resistance.

**Results:**

Using this newly developed core model, the evolution of three cell types: normal, tumor, and drug-resistant tumor cells, is studied through a series of numerical simulations. In addition, we illustrate critical factors that cause different dynamical patterns for normal and tumor cells. Among these factors are the co-dependency of the normal and tumor cells, the cells’ response mechanism to a single or multiple chemotherapeutic treatment, the drug administration sequence, and the treatment starting time.

**Conclusion:**

The results provide us with a deeper understanding of the possible evolution of normal, drug-responsive, and drug-resistant tumor cells during the cancer progression, which may contribute to improving the therapeutic strategies.

## Introduction

Assessing the evolution of cancer, in the presence of surrounding normal cells, is the subject of many biomedical studies. Recently reported evidence strongly indicates that the dynamics of tumor cells and the surrounding normal cells are not independent of each other and may be mutually tuned [1–8]. Examination of the coupled population dynamics of tumor and normal cell populations can potentially provide substantial knowledge that may contribute to the identification of more effective therapeutic interventions, particularly in aging populations. Among the variety of research papers in this field, many are based on the analysis of mathematical and computational models. In many of these models, the growth of normal and tumor cells are considered to be independent and are expressed by such functions as the Gompertz, the logistic, and the exponential equations [9–13]. However, the mutual interaction of tumor cells with surrounding normal cells, which was first mathematically introduced in a conjoint model by Witten [14], could shed light on some of the complex patterns that can be detected during cancer progression [15–17].

The interaction of tumor and normal cells is not the exclusive factor causing different dynamical patterns during cancer progression, The interaction of cells with the host immune system, therapeutic agents such as chemotherapy, immune therapy, or any other therapeutic interactions are additional factors which can influence the evolution patterns of the cell populations [18–33].

While researchers continuously improve cancer treatment strategies, one of the most serious obstacles in cancer treatment are related to drug resistance, where the chemotherapeutic treatments do not lead to the hoped for outcome. The issues related to the drug resistance have been broadly studied from a variety of different perspectives [34–38].

This work aims to contribute to a deeper understanding of drug resistance effects on cancer progression through the analysis of a new mathematical model and its concomitant computational simulation for a coupled tumor-normal cell framework that is more aligned with experimental evidence. To simulate the population evolution of our model, we have used Mathematical V7.0. Model parameter values are estimated based on values previously introduced in the literature and are given in Table 1 of this paper. Additionally, the other parameters in some parts are varied in order to study the system’s evolution.

**Table 1:** **Table of parameters: parameters used in simulations have been estimated based on the values introduced in following sources**

Parameter	Units	Description	Estimated value	Reference source
r_T_	Time^−1^	Growth rate for the drug sensitive tumor cells	0.3	[31]
K_T_	Cells	Carrying capacity of tumor cells	1.2×10^6^	[31]
β	Time^−1^	Normal-tumor cell interaction rate	1	[31]
ρ_0_	Cells	Interaction clearance term	1	[31]
ρ_1_	Cells	Half-saturation for interaction	1000	[31]
r_N_	Time^−1^	Growth rate for the normal cells	0.4	[31]
K_N_	Cells	Carrying capacity of normal cells	10^6^	[31]
κ	Time^−1^	Tumor-normal cell interaction rate	0–0.028	[31]
T^*^	Cells	Critical size of tumor	3×10^5^	[31]

This paper is structured as follows: in The basic conjoint tumor-normal cell model Section, we briefly review the normal-tumor cell conjoint model. In Conjoint core model in a chemo-resistance setting section, we introduce the drug resistance assumptions and subsequently modify the conjoint model to make a distinction between tumor-responsive and tumor-resistant cells. In Chemo-treatment strategies in a resistance setting section, we include the effects of chemotherapeutic treatment to the modified conjoint model and we examine and discuss the dynamics of the system. We conclude and examine future research directions.

### The basic conjoint tumor-normal cell model

**Figure 1:**
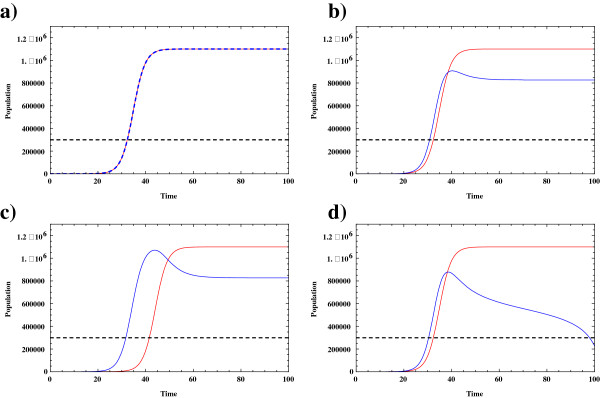
**Blue curve: Evolution of normal cells. Red curve: Evolution of tumor cells.** In this figure the blue curve illustrates the evolution of the normal cell population and the red curve illustrates the evolution of tumor cell population. The horizontal dashed line represents the magnitude of the critical population of tumor cells T^*^; **a)** In this figure, the normal and the tumor cells grow, uncoupled following a Gompertzian law. *K*_*T*_ *= K*_*N*_ = 1.1*10 ^*6*^ *; r*_*T*_ *= 0.4; r*_*n*_ *= 0.4;T* ^*^_C_ = 3*10 ^*5*^; **b)** In this figure, the normal and the tumor cells grow conjointly using the following parameter values *β = 1;ρ*_*0*_ *= 1;ρ1 = 1000; κ =0.028*. In this case, the tumor cells can now suppress the growth behavior of normal cells. The population of the normal cells declines as the population magnitude of the tumor cells passes the critical value of *T* ^*^_C_ = 3*10 ^*5*^ (the horizontal dashed line). The inhibition time in which the normal cells begin to decrease is approximately *t = 30* (unit of time); **c)** *β = 50*. In this simulation the role of normal cells on the growth of tumor cells is significantly increased. As can be seen, the growth of tumor cells starts with a delay. As compared with the Figure 1 **b**, the shrinkage starts at almost *t = 40*. Therefore, the normal cells maintain a higher population for a longer time. Figure 1 **d**, expresses the evolution of normal and tumor cells when *κ =0.039, β = 1*. This time, the interaction effect of tumor cells on normal cells is increased. Under this set of simulation conditions, the population of normal cells goes to minimum value and they die out of system.

Feizabadi & Witten [28] extended the earlier work of Witten [17] proposing the following generalized model to describe the inter-connection between normal and tumor cells. The core model equation system is given by:


where *T(t), N(t), K*_*T*_*, K*_*N*_*, r*_*T*_*, r*_*N*_ are respectively the total number of tumor cells at time *t*, the total number of normal cells at time *t*, the carrying capacity for the tumor cells, the carrying capacity for the normal cells, and the per capita growth rate for the tumor and normal cells, and *f*_*T*_(*N*), *f*_*N*_(*T*) are the functional rules relating normal-to-tumor and tumor-to-normal interaction respectively [39]. Witten and Feizabadi [28] have shown that one possible set of coupled, nonlinear equations for the tumor-normal cell system may be expressed follows:


where *T, N, K*_*T*_*, K*_*N*_*, r*_*T*_*, r*_*N*_ are previously defined. In each equation, the second terms represent the interaction between tumor and normal cells. Here, *β* and *κ* have the units of 1/time. Also, for consistency, *ρ*_*0*_ and *ρ*_*1*_ have units of cells. T* is the critical size of the tumor and as the size of tumor exceeds the critical size, the normal cells growth rate decreases. Figure 1(a) illustrates the time evolution of normal and tumor cells in a hypothetical environment in which they grow independently (uncoupled) from one another, and where each cell population follows a Gompertzian-like behavior. In this figure the growth parameters are considered to be identical for both normal and tumor cells. In Figure 1(b) and 1(c), the conjoint growth is added to the model. As can be seen by the different parameter values, the growth of the normal and tumor cells can be affected as a result of the cellular interactions. The ability of tumor cells to inhibit the normal cell’s growth increases as the population of tumor cells passes the critical value *T*. In Figure 1(c) the growth of tumor cells is delayed due to the influence of the surrounding normal cells introduced by a higher value for the interaction parameter *β*. Figure 1(d) illustrates the case in which the normal cells have died out as a result of the strong interaction effect from the tumor cells. These results are not surprising as this is essentially a competitive exclusion model.

In the next portion of the paper we consider a modified conjoint model in which we make a distinction between the group of tumor cells that are responsive to one type of chemotherapeutic agent and those which are resistant to that same chemotherapeutic agent.

## Mathematical models and results

### Conjoint core model in a chemo-resistance setting

One of the ongoing challenges to maximizing chemotherapeutic success in cancer treatment is the long-standing challenge of tumor cell resistance to single or multiple drug cocktails [40]. This mechanism, known as chemo-resistance, is complex and depends upon many factors including but not limited to the specific drug, specific tumor, or the specific host’s defense mechanism [41]. Coupled with chemo-resistance is the challenge of age-related sensitivity or insensitivity to various drug cocktails. Thus, a dose that might not be lethal in a 20 year-old patient could well be lethal in a 60 year-old patient.

Drug resistance is classified into three major categories. The first category is associated with pharmacologic resistance or when the drug cannot effectively reach the tumor site due to insufficient pharmo-kinetic properties. The second category is rooted in the biochemistry of the tumor cell, for example when the drugs are not active at the tumor cell sites. The third category is when chemo-resistance results from genetic mutation of the tumor cells [42, 43].

In order to overcome drug resistance, we need to improve treatment efficacy by better understanding the resistance mechanisms and their effect on the cancer progression. This is a complex challenge and, so far has remained beyond traditional clinical and experimental examination. The complexity of the problem has led investigators to further develop their understanding using modeling and simulation methods. In fact, this challenge has been the subject of many theoretical and computational studies [44–51]. In the upcoming section of the paper we focus on this problem by introducing a chemo-resistant tumor cell component to our model. We modify the model as follows.

**Figure 2:**
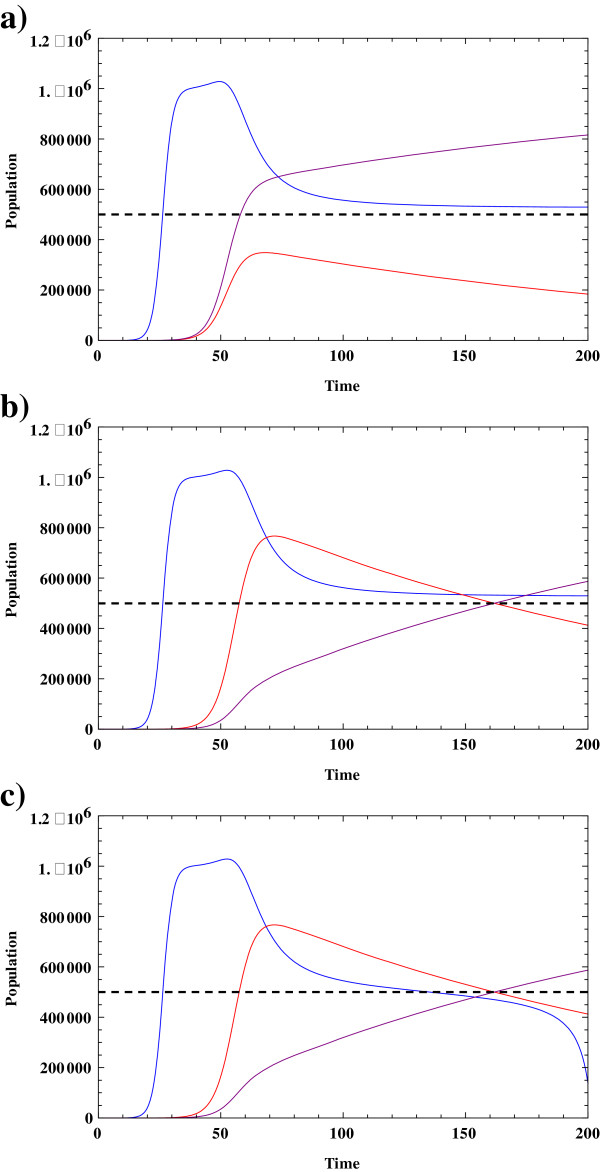
**The evolution of normal cells and tumor cells in a chemo-resistance setting.** In this figure the blue curve illustrates the evolution of normal cell population, red curve illustrates the evolution of the drug responsive tumor cell population, and the purple curve illustrates the evolution of the drug resistant tumor cell population. The horizontal dashed line represents the magnitude of the critical population of tumor cells, T^*^. **a)** In this figure, normal and tumor cell populations grow in a coupled setting where the parameter values are given by. *K*_*T*_ *= K*_*N*_ *= K*_*R*_ *= 10* ^*6*^ *; r*_*T*_ *= r*_*r*_ *= 0.25; r*_*n*_ *= 0.4; T* ^*^_C_ = 5*10 ^*5*^ ; κ = 0.124; μ = 5*10 ^*−3*^ *.* Due to mutation and growth, the population of drug resistant tumor cells is higher than that of the drug sensitive tumor. Normal cell numbers decrease as the total number of tumor cells exceed the magnitude of the critical tumor cell population. **b)** In this figure, *r*_*T*_ *= 0.25; r*_*r*_ *= 0.2.* Here, the population of drug responsive tumor cells is higher at the beginning of the developmental curve. However, at approximately t = 160 days, the system contains a higher population of drug resistance cells. **c)** In this figure, *κ = 0.126, r*_*T*_ *= 0.25; r*_*r*_ *= 0.2.* Using this set of parameters we find that the population of normal cells has become smaller that critical value of tumor cells. In such a case the normal cells die out of the system.

First, we rely on reported evidence indicating that metastatic tumors with larger sizes are more likely to show resistance to chemotherapeutic drugs [52, 53]. Therefore, the effect of normal cells in reducing the large population of tumor cells is not significant. Consequently, we have assumed that the second term is equation 2a is ignorable. For the purposes of discussion, we have assumed that the drug resistant tumor cells are created as a result of tumor cell mutation. They become resistant tumor cells with a mutation rate *μ*[42]. Further, we assume that the drug resistant tumor cells also grow under the logistic growth law where the population growth rate, r_R_, is modified by the density dependent term associated to the total number of tumor cells. In our model, *K*_*R*_ is the carrying capacity for the drug resistant tumor cells. Lastly, we remember that the population of the normal cells is controlled by the tumor cell population. Combining all of these assumptions along with our earlier model equations, we obtain the following new equation system:


The behavior of the normal, drug sensitive tumor, and drug resistant tumor cells is simulated in Figure 2. Understanding the evolution of each component becomes more critical in connection with the treatment of the system with chemotherapeutic agents that will be discussed in the next section.

In the drug resistance model, as the population of total tumor cells which now includes both the responsive and the resistant tumor cells, passes the critical value T^*^ the normal cell population decreases in number. In Figure 2(a) the growth rate of both the drug responsive and the resistant tumor cells are considered to be identical. In this case the population of the resistant tumor cells is larger due to the fact that mutation of responsive tumor cells continually decreases the population of the responsive tumor cell population and subsequently increases the size of the resistant tumor cell population. In Figure 2(b), the growth rate of responsive tumor cells is higher than that of the resistant tumor cells. Here, we can see that for a period of time, the population of the responsive tumor cell population is larger. However, ultimately the population of the drug resistant tumor cells becomes higher than that of the tumor responsive cells. In Figure 2(c), the tumor-normal cell coupling coefficient is increased slightly. Under this new condition, the population of normal cells has become smaller than the critical value, T^*^ = 5*10^5^. Therefore, the tumor cells overwhelm the normal cells and the normal cells die out of the system faster than before, In addition, the population of the drug resistant tumor cells continues to grow. We next consider the effect of adding a chemotherapeutic agent to our system.

### Chemo-treatment strategies in a resistance setting

The conjoint model, in the presence of the chemo-resistant tumor cells, may also be modified to consider the introduction of chemotherapeutic agents. To systematically investigate the evolution of the cells, we have simulated the system’s dynamics under the following conditions. We first assume that due to the drug resistance, the first chemotherapeutic agent introduced to the system has a cytotoxic effect only upon the drug responsive tumor cells, *T*. Due to the effect of this toxicity, the population of tumor cells decreases following an interaction with this drug. Secondly, we have simulated the dynamics of the system under a combination therapy, where the second chemotherapeutic agent is effective only on the drug resistant tumor cells, *T*_*R*_*.* Finally, the effects of the drug cocktail are studied when the time of drug administration is varied.

**Figure 3:**
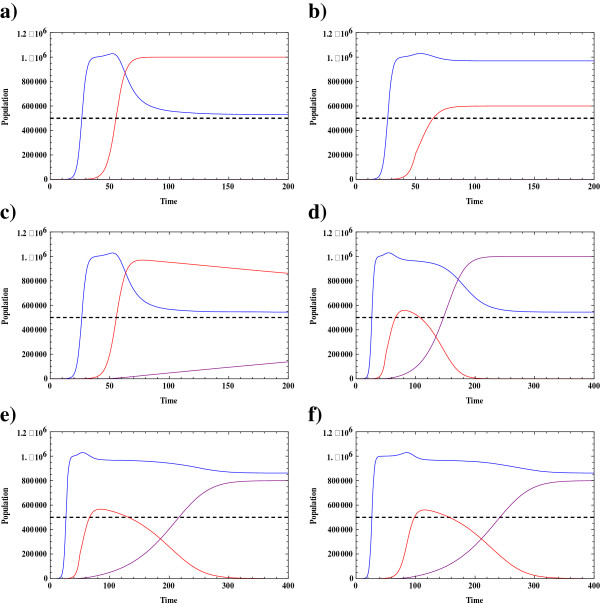
**The evolution of normal and tumor cells during the phase of therapy in a chemo-resistance setting.** In this figure the blue, red, and purple curves illustrate the evolution of the normal population, the drug responsive tumor cell population, and the drug resistant tumor cell population respectively. **a)** The behavior of the coupled normal-tumor cells in the absence of drug resistant tumor cells and with no chemotherapy is simulated when *K*_*T*_ *= K*_*N*_ *= 10* ^*6*^ *, r*_*T*_ *= 0.25 r*_*n*_ *= 0.5, κ = 0.124, T* ^*^_C_ = 5*10 ^*5*^. **b)** The conjoint normal-tumor cells are now simulated in the presence of an anti-tumor drug. The fraction killing rate is considered to be constant with the value of *a*_*T*_ *(1-e* ^*-MC*^ *) = 0.1*, and the treatment is started at *t = 50* days. It is assumed that the administered drug has no effect on normal cells*; a*_*N*_ *(1-e* ^*-MC*^ *) = 0*. **c)** the drug resistance conjoint model in which the tumor cells are categorized either as drug responsive or drug resistant is simulated when *K*_*T*_ *= K*_*N*_ *= K*_*R*_ = 1*10 ^*6*^ *, r*_*T*_ *= 0.25 r*_*n*_ *= 0.5, r*_*R*_ *= 0.1, κ = 0.124, T* ^*^_C_ = 5*10 ^*5*^ *, μ = 10* ^*−3*^. **d)** The three component model is simulated as the system interacts with an anti-tumor drug effective only on the drug responsive tumor cells, *a*_*T*_ *(1-e* ^*-MC*^ *) = 0.1* at *t = 50* days. **e)** In this figure, the dynamics of the three-component is simulated where the system is treated with two distinct drugs, one effective only on drug responsive tumor cells and one on the drug resistant tumor cells when *a*_*T*_ *(1-e* ^*-MC*^ *) = 0.1* and *a*_*R*_ *(1-e* ^*-MC*^ *) = 0.02* and both treatments are started at *t = 50*. **f)** A similar treatment to that of Figure e is simulated at *t = 5* days.

As suggested by Gardner [54] and used in other studies [29, 33], the drug interaction may be structured as *a*_*ϕ*_(1 − *e*^− *MC*^)*ϕ*. Here, φ is the cell population number of the three types of cells: *T, T*_*R,*_*N.* The parameter *C* is the concentration of the drug at the site, M is the pharmacokinetic factor, and a_φ_ is the response factor. The function *F*(*C*) = *a*_*ϕ*_(1 − *e*^− *MC*^) is the fraction cell kill for a given amount of drug “C”. In the presence of the chemotherapeutic agents, the previous mutation model may now be modified as follows:


In the first two simulations, Figure 3(a,b), the conjoint tumor-normal cell population model is simulated where there is no mutation and hence no resistant tumor cell population. In a drug-free system, the coupling effect and the decrease in of normal cells can be observed as the tumor cells exceed the critical value T*. This same system is then simulated when the tumor cells interact with anti-tumor drugs and the toxicity of the drug kills the tumor cells. As the size of tumor cells decrease, the normal cells recover and subsequently return to a higher population number. In Figure 3(a-f), the number of normal cells increases slightly beyond the carrying capacity (*K*_*R*_ = 1*10^*6*^*).* At this point which, for instance, can be seen around t = 50 in Figure 2(b), the tumor cell population is less than the magnitude of the critical population, T^*^; therefore, the second term of equation 4 is positive and slightly increases the population of normal cells. In fact, normal cells react to the presence of small groups of tumor cells in the system.

In Figure 3(c), tumor cells we return to the two population tumor cell model; resistant and non-resistant tumor cells. The appearance of the drug resistant tumor cells at t ~ 50 days and their subsequent growth is illustrated in this figure. Given the mutation rate of μ = 10^−3^, the population of drug responsive tumor cells decreases. However, the population of drug resistant tumor cells increases over time as there are no chemotherapeutic agents that target this population.

Figure 3(d) illustrates the dynamics of the cell populations when the system interacts with an anti-tumor drug which is effective only on the drug responsive tumor cells. The drug is administered at *t = 50* days. The drug responsive tumor cells decrease and die out of the system. Due to the chemotherapeutic treatment, the total number of tumor cells falls below the critical size of tumor cells. Therefore, the normal cells maintain higher population for a period of time. However, mutated drug-resistant tumor cells increase and their population will eventually pass the critical value. As a result, the normal cells start to decrease again. This kind of chemotherapeutic intervention can create a delay in possible organ failure by maintaining a higher number of normal cells for a period of time.

In the next simulation, we introduce the combination therapy protocol. A combination therapy is considered a more effective treatment strategy with cancers that show resistance to some of the chemotherapeutic agents. In this mode of intervention, while the tumor is treated by the recommended chemotherapeutic drug protocol, other chemotherapeutic drugs are also used in order to target those tumor cells that have developed defense mechanisms against the first type of chemotherapeutic agent. In Figure 3(e) illustrate the dynamics of the system under a multiple therapeutic protocol. In this simulation, both drugs are administered at the same time, t = 50. A lower dosage and therefore lower toxicity is considered for the anti-resistant tumor cell population. This mode of intervention was chosen due to the fact that, at the start of chemotherapy, drug responsive tumor cells have a higher population. Therefore, a higher drug dosage was considered for the non-resistant population. As can be seen in Figure 3(e), the normal appear, at first, to be stimulated by the tumor cell population growth but eventually return to their carrying capacity value while the drug sensitive tumor cell population dies out of the system as a result of interaction with the anti-cancer drug. In this simulation, due to the toxicity of the second type of the introduced anti-cancer drug, the maximum population of the drug resistant tumor cells is much smaller as compared to a case in which the tumor is treated with only a single chemotheraputic drug (Figure 3(d)).

In Figure 3(f), both treatments are supposed to be started simultaneously at an earlier time; t = 5 days. As can be seen in this figure, not only have the normal cells reached their carrying capacity, but also the growth of tumor cells has been delayed.

## Conclusions

This work, a modification of our previous work, focuses on examining the dynamics of interconnected normal and tumor cells treated with chemotherapeutic agents, when some of the tumor cells show chemo-resistance. We examined these dynamics using a collection of different simulation parameters. Simulations demonstrated that in a conjoint system, normal cells enter a phase of diminished growth as the total number of tumor cells passes the magnitude of a critical tumor cell population . To control the population of tumor cells and the decrease of the population of normal cells, which may lead to organ failure, tumor cells can be treated with chemotherapeutic agents. In order to overcome the drug resistance, implementing a combination treatment is recommended. In a combination therapeutic approach, the dosage and the time of chemotherapy introduction play a critical role in minimizing the population of tumor cells, while maintaining the maximum population of the normal cells. According to our simulations, starting the combinatory therapy in the early stage of the cancer progression may lead to better control of the cancer progression as this treatment protocol can minimize the tumor cell population.

In our simulations, the growth rate and mutation rate of the cells are two other important factors that can potentially cause different evolution patterns. Another factor that plays a significant role in the system dynamics is the dosage of the anti-tumor drugs. It is more probable that a better response is achieved by increasing the drug dosage. However, since a majority of the chemotherapeutic drugs are toxic to normal cells and the host immune system, Consequently, the dosage and the level of toxicity must be carefully considered in order to minimize the potential damage to normal cells and to the patient.

In addition, damages that can be produced by chemotherapy are significant in the presence of an impaired immune system. The lack of inclusion of the effects of the immune system in our model is one of its limitations. Therefore, considering how the interaction of tumor cells with the host immune system may affect tumor progression are elements that can potentially be included in our model to achieve outcomes more aligned with clinical and biological observations.

## Methods

### Computational calculations

All calculations were executed on an PC using Mathematica v7.0. Code is available from the first author.
